# Supplementary material for: Chi hotspot control of RecBCD helicase-nuclease by long-range intramolecular signaling
Source: Sci Rep. 2020 Nov 5;10:19415. doi: 10.1038/s41598-020-73078-0 (PMC7644769; doi:10.1038/s41598-020-73078-0)
Supplement: Supplementary file 1 — Supplementary Information. [file 41598_2020_73078_MOESM1_ESM.pdf]

Chi hotspot control of RecBCD helicase-nuclease by long-range intramolecular signaling

SK Amundsen, AF Taylor, and GR Smith

Supplementary Tables and Figures

Supplementary Table S1. Mutants altered in RecC-RecD contact point (CD)

Allele number[a]	Alternate allele designation[a]	C2[b] 541-544 QGEW	D2[b] 97-99 PTP	Genetic assays	
				Chi hotspot activity[c]	*E. coli* Hfr cross (relative recombination frequency)[d]
WT		. . . .	. . .	5.1 ± 0.09	1.0
C2820	C2△	△△△△	. . .	1.5, 1.4	1.0, 0.64
C2821	C2ala	AAAA	. . .	3.2, 3.5	0.78, 0.67
D2824	D2△	. . . .	△△△	0.9, 1.0	0.43, 0.29
D2825	D2ala	. . . .	AAA	2.4, 2.9	0.38, 0.62
C2821 D2825	C2ala D2ala	AAAA	AAA	2.2, 2.3	0.66, 0.76
C2820 D2824	C2△ D2△	△△△△	△△△	1.4, 1.1	0.81, 0.49

[a] The indicated mutation was on a derivative of plasmid pSA607 (*recBCD*$^+$) in strain V2831 (Δ*recBCD2731*). Alternate allele designations are those used in the main text.

[b] The contact point is composed of the indicated amino acids at the indicated positions. The alleles have mutations deleting the indicated amino acid (△); substitutions (when made) contain the indicated amino acid; (.) indicates that the amino acid is wild type.

[c] Chi hotspot activity in lambda vegetative crosses was determined as described in Materials and Methods [31]. Chi hotspot activity = $\sqrt{(t/c)_1/(t/c)_2}$ where t/c is the ratio of turbid (*cI*$^+$) to clear (*cI857*) recombinant plaques among $J^+ R^+$ recombinants in cross 1 (with χ^+*D123*) and cross 2 (with χ^+*76*).

[d] Frequency of His$^+$ [Str^R] recombinants per viable Hfr parent relative to that in the concurrent *recBCD*$^+$ cross. Wild-type frequency was 6.99% ± 0.46% (n = 25). The relative recombinant frequency for a null mutant (*recB21*) is 0.005.

Supplementary Table S2. Mutants altered in RecD-RecB contact point (DB)

Supplementary Table S2A.

Allele number[a]	Alternate allele designation[a]	D3[b] 521-528 SVQPSRLP	Genetic assays	
			Chi hotspot activity[c]	*E. coli* Hfr cross (relative recombination frequency)[d]
WT		....	5.1 ± 0.09	1.0
D2826		..E.DE..	3.5, 2.9	1.08, 0.94
D2827		...A....	4.9, 4.4	1.06, 0.87
D2828		......A.	3.7, 3.2	0.76, 0.69
D2829		AA......	3.4, 2.8	0.58, 0.83
D2830		AA....A.	1.5, 1.3	0.52, 0.39
D2831	D3ala	..AAAA..	1.9, 1.4	0.41, 0.49
D2832		AAAA....	2.8, 2.6	0.87, 0.76
D2833	D3△	..△△△△..	1.0, 1.2	0.24, 0.24
D2834		..△△....	4.0, 4.7	1.26, 0.87
D2835		....△△..	5.1, 4.8	1.21, 0.69
D2836		△△......	0.96, 1.5	0.24, 0.46
D2837		......△△	3.9, 3.7	0.79, 0.63
D2838		△△△△....	0.9, 1.1	0.39, 0.46
D2839		..△△..△△	1.1, 1.2	0.23, 0.31
D2840		....△△△△	0.9, 0.9	0.91, 0.79
D2841		△△..△△..	1.4, 1.2	0.31, 0.46
D2842		△.......	3.1, 3.2	0.67, 0.77
D2843		.△......	1.6, 1.4	0.42, 0.61

Footnotes are as in Supplementary Table S1.

Supplementary Table S2B.

Allele number[a]	Alternate allele designation[a]	B3[b] 634-646 DEHAWDVVVEEFD	Genetic assays	
			Chi hotspot activity[c]	*E. coli* Hfr cross (relative recombination frequency)[d]
WT		....	5.1 ± 0.09	1.0
B2862		.A...........	4.9, 4.8	0.87, 0.78
B2863		.A...A.......	4.1, 3.9	0.76, 0.72
B2864		.A...A...A...	3.8, 3.9	0.84, 0.79
B2844		.A...A...A..A	2.1, 2.2	0.31, 0.23
B2845	B3ala	AA...A...AA.A	1.8, 1.6	0.21, 0.34
B2846		.........Q..S	3.6, 3.1	0.91, 0.78
B2847		.R...N...QK.S	2.9, 3.0	0.83, 0.68
B2848		KR...........	4.9, 4.8	0.92, 0.94
B2849		KR.......RR..	1.5, 2.1	0.48, 0.39
B2858		.........RR..	4.3, 3.9	0.39, 0.54
B2850		KR.......RR.K	1.7, 1.5	0.42, 0.33
B2851	B3lys,arg	KR...K...RR..	1.2, 1.4	0.39, 0.42
B2852	B3∆	∆∆∆∆∆∆∆∆∆∆∆∆∆	0.9, 1.0	0.006, 0.009
B2853		.....∆∆∆∆∆...	4.1, 4.7	0.87, 0.93
B2854		∆∆∆∆∆........	4.9, 3.5	0.93, 0.68
B2855		..........∆∆∆	3.8, 3.6	0.29, 0.38
B2856	B3∆(10)	∆∆∆∆∆∆∆∆∆∆...	4.6, 4.2	0.88, 0.78
B2857		.....∆∆∆∆∆∆∆∆	3.1, 2.7	0.08, 0.07
B2859		.........AAAA	4.8, 4.9	0.68, 0.84

Supplementary Table S2C.

Allele number[a]	Alternate allele designation[a]	B3[b] 634-646 DEHAWDVVVEEFD	D3[b] 521-528 SVQPSRLP	Genetic Assays	
				Chi hotspot activity[c]	*E. coli* Hfr cross (relative recombination frequency)[d]
WT		....	....	5.1 ± 0.09	1.0
B2845 D2830	B3ala D3ala	AA...A...AA.A	AA....A.	1.5, 1.4	0.48, 0.39
B2845 D2833	B3ala D3∆	AA...A...AA.A	..∆∆∆∆..	1.1 ± 0.06	0.53, 0.34
B2844 D2833		.A...A...A..A	..∆∆∆∆..	1.8, 1.6	0.42, 0.38
B2847 D2826		.R...N...QK.S	..E.DE..	1.8 ± 0.2	0.66 ± 0.1

Footnotes are as in Supplementary Table S1.

Supplementary Table S3. Mutants altered in or near RecB-RecC contact point (BC)

Allele number[a]	Alternate allele designation[a]	B4[b] 913-922 GHGIAQDLMP	C4[b] 599-608 FLPDAETEAA	D4[b] 469-475 HRHPHSR	Genetic assays: Chi hotspot activity[c]	Genetic assays: *E. coli* Hfr cross (relative recombination frequency[d])
WT		....	....	....	5.1 ± 0.09	1.0
B2865	B4ala	AAAAAAAAAA	....	....	3.6, 3.7	0.58, 0.41
B2860	B4△	△△△△△△△△△△	....	....	4.2, 4.3	0.81, 0.52
C2861		....	...A.A.A..	....	4.9, 3.8	0.87, 0.68
C2823	C4△(7)	....	...△△△△△△△	....	2.3 ± 0.12	0.32 ± 0.03
C2822	C4△	....	△△△△△△△△△△	....	1.1 ± 0.12	0.24 ± 0.04
D2867		....	....	AAAAAAA	4.4 ± 0.15	0.74 ± 0.06
D2868		....	....	△△△△△△△	3.9 ± 0.17	0.83 ± 0.07
B2860	B4Δ	△△△△△△△△△△	....	....	4.2, 4.3	0.81, 0.52
B2860 D2868		△△△△△△△△△△	....	△△△△△△△	5.2, 4.3	1.31, 0.59
B2865 D2868		AAAAAAAAAA	....	△△△△△△△	4.0, 4.6	0.22, 0.29
C2822	C4Δ	....	△△△△△△△△△△	....	1.1 ± 0.12	0.24 ± 0.04
C2823	C4Δ(7)	....	...△△△△△△△	....	2.3 ± 0.12	0.32 ±0.03
B2865 C2861		AAAAAAAAAA	...A.A.A..	....	2.5 ± 0.26	0.31 ± 0.09
C2861 D2868		....	...A.A.A..	△△△△△△△	3.9 ± 0.14	0.53 ± 0.08
B2860 C2822	B4Δ C4Δ	△△△△△△△△△△	△△△△△△△△△△	....	1.1, 1.3	0.09, 0.22
B2865 C2861 D2868		AAAAAAAAAA	...A.A.A..	△△△△△△△	3.4 ± 0.27	0.44 ± 0.06

Footnotes are as in Supplementary Table S1.

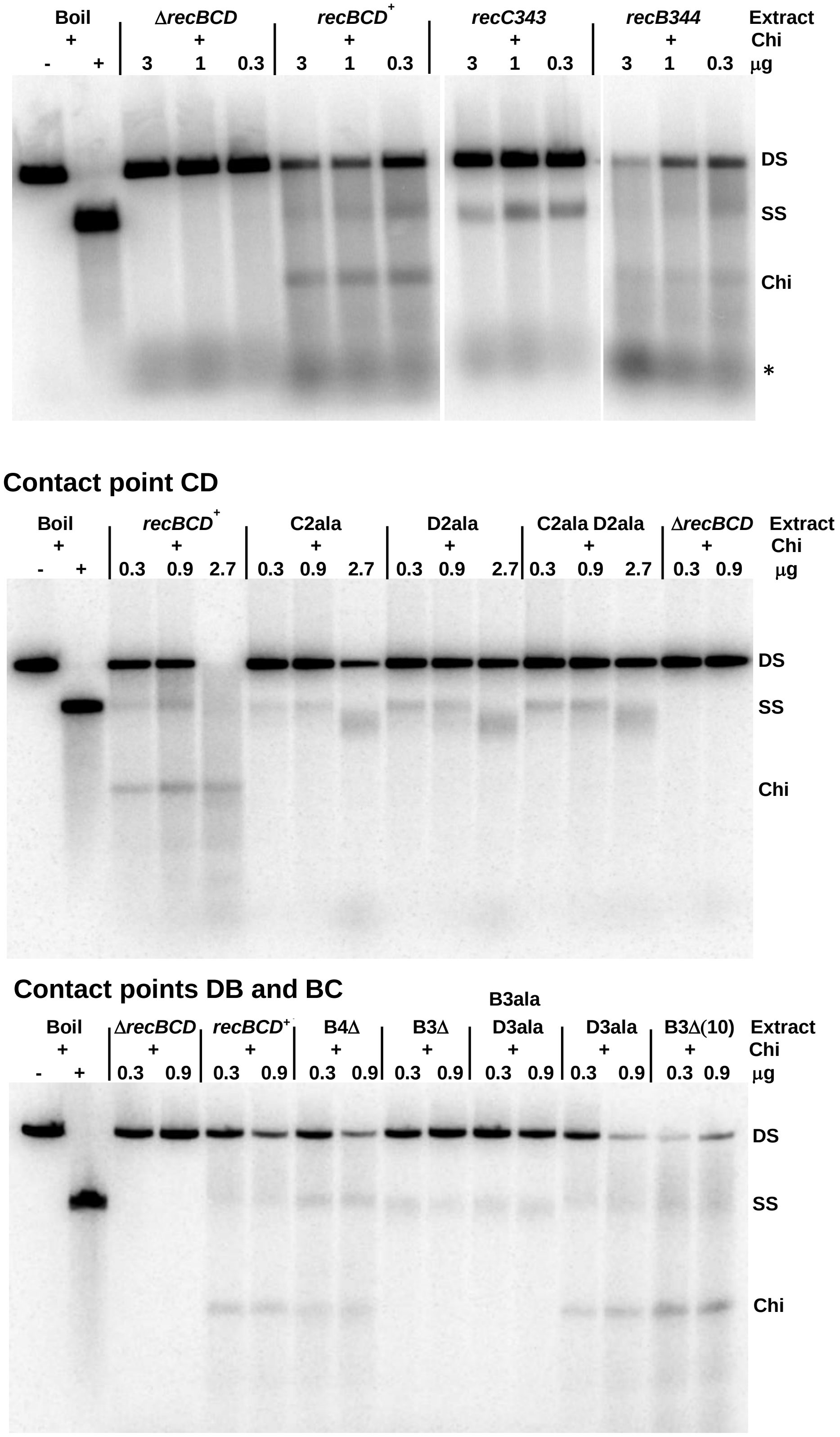

Supplementary Figure S1. RecBCD contact-point mutants, *recC343,* and *recB344* retain DNA unwinding activity but have reduced or undetectable cutting of DNA at Chi hotspots. Extracts of the indicated mutants were assayed for unwinding and cutting of linear pBR322 DNA (4.3 kb long) with a Chi site (χ^+*F225*) 1470 bp from the 5' [^{32}P]-labelled DNA end. Note that the Chi-cut species and ss DNA are reaction intermediates and their observed amount is not necessarily a linear function of enzyme amount. ds substrate (DS), unwound ss DNA (SS), Chi-cut DNA (Chi) and limit digestion products (oligonucleotides; *) are indicated. In the top gel, some lanes were removed, as indicated by the white spaces to the left and right of the *recC343* samples (see Fig. S7).

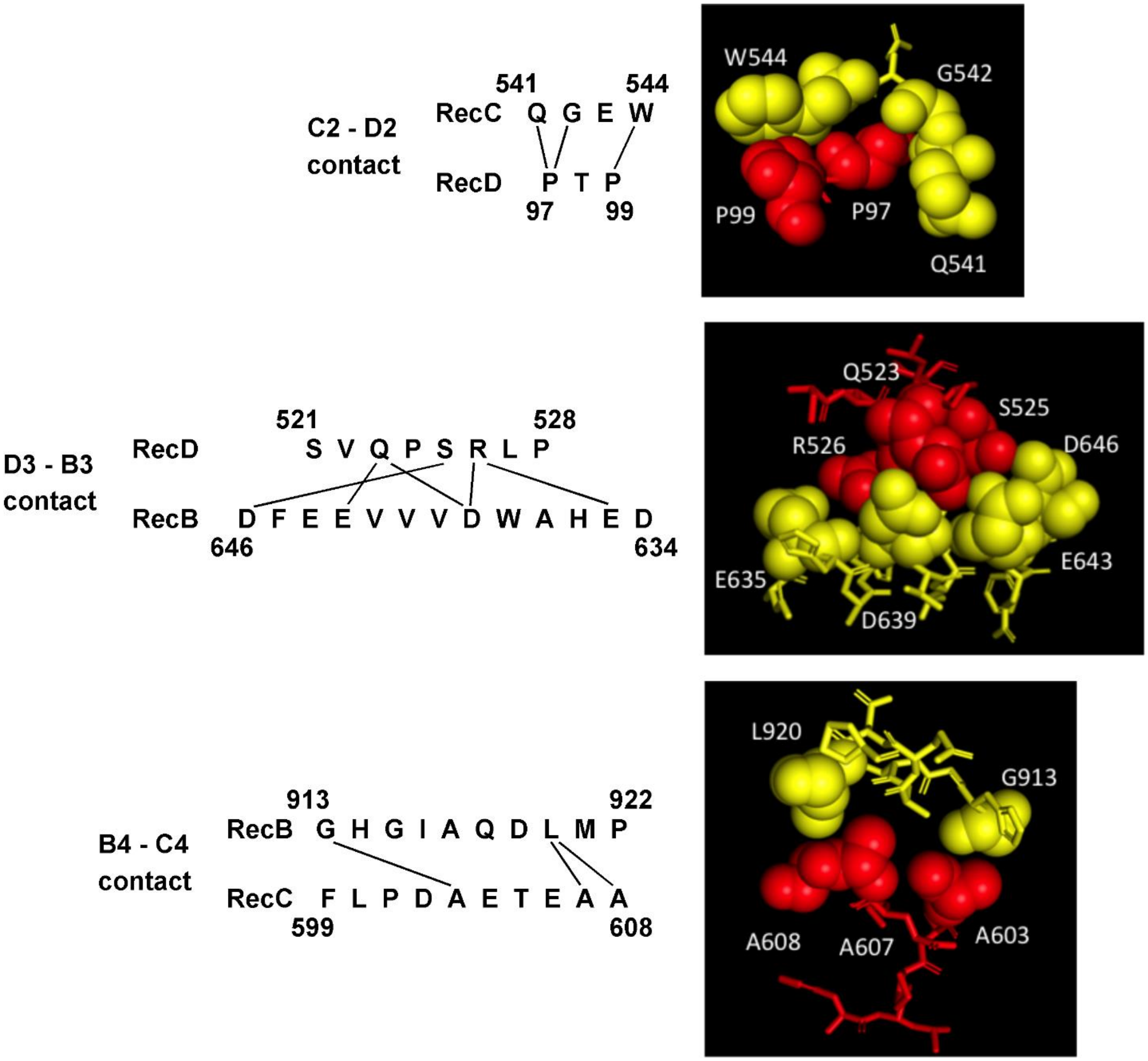

Supplementary Figure S2. **Amino acids in close proximity at each contact point.** The amino acid sequences of each side of each contact point are shown; note that contact point B3 reads from right to left (amino to carboxyl ends). Lines indicate amino acids on each side of the contact point that have atoms in close proximity (less than about one atom's diameter). Images on the right show the amino acids noted on the left. Amino acids in spheres are the amino acids at the ends of each connecting line on the left; other amino acids are in stick representation. C2, B3, and B4 are yellow, and D2, D3, and C4 are red, as in Fig. 2.

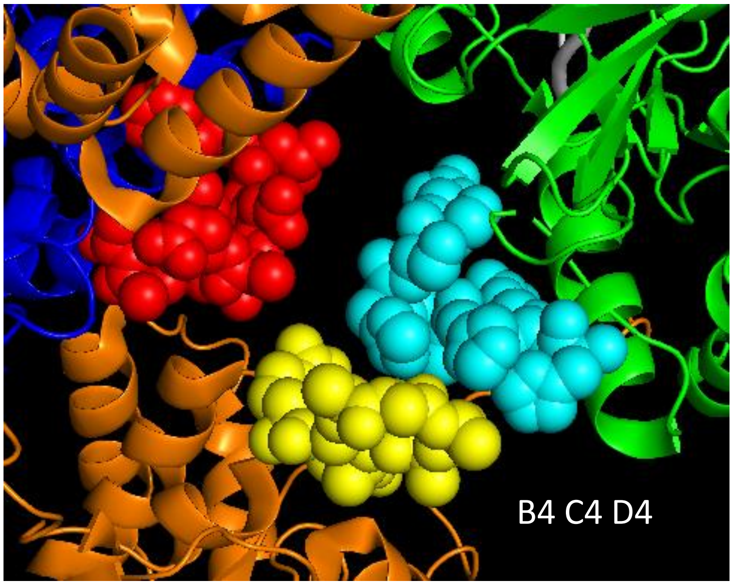

Supplementary Figure S3. Contact point D4, close to contact points B4 and C4. Contact points are shown as spheres. B4 (amino acids GHGIAQDLMP at positions 913 – 922, modeled as alanines, of RecB) is yellow, C4 (amino acids FLPDAETEAA at positions 599 – 608 of RecC) is red, and D4 (amino acids HRHPHSR at positions 469 – 475 of RecD) is cyan. Shown is part of the cryoEM structure PDB 5LD2.

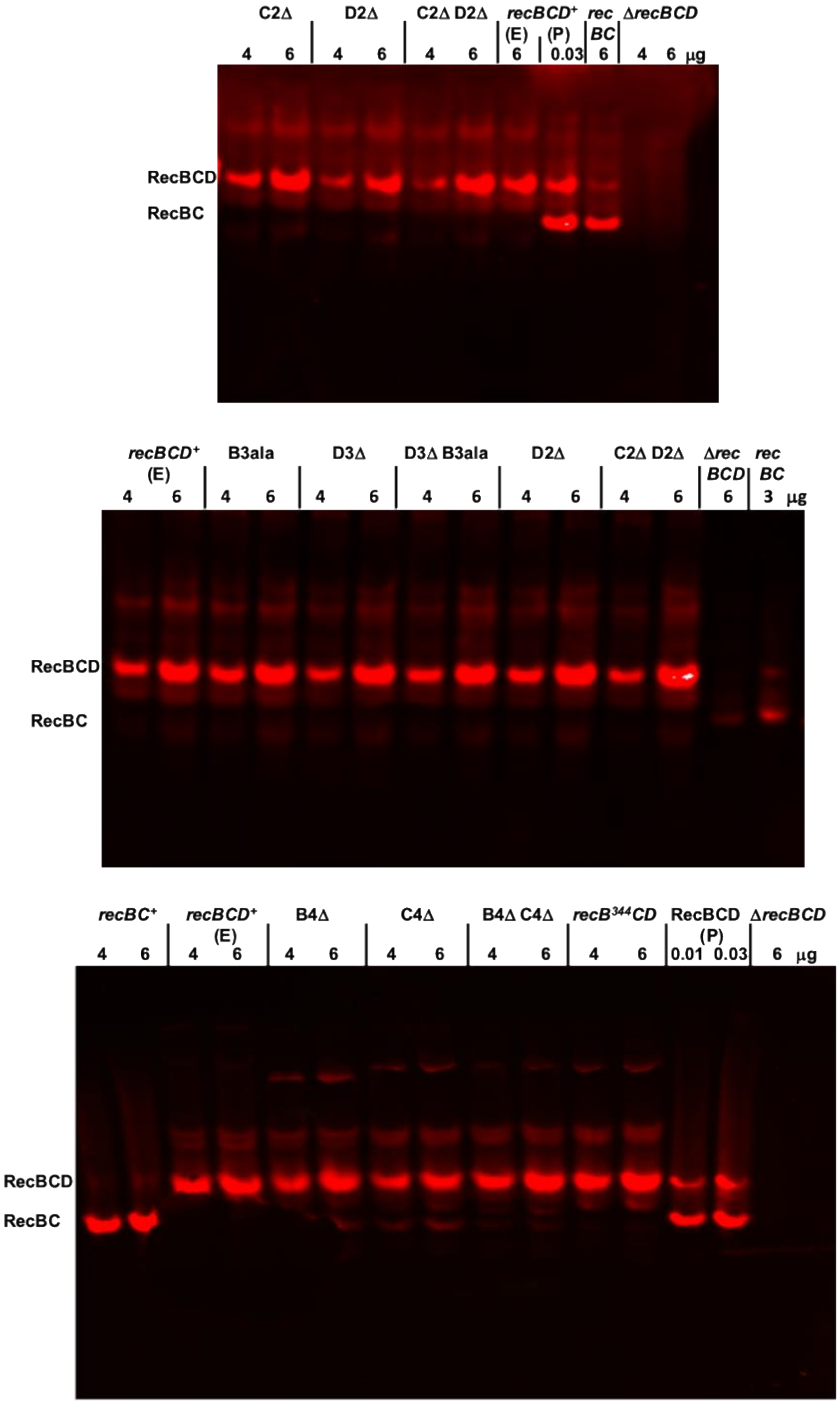

Supplementary Figure S4. Signal transduction mutants retain wild-type levels of assembled heterotrimeric RecBCD enzyme. Extracts of the indicated mutants were analyzed by native gel electrophoresis followed by transfer to a membrane. RecBCD enzyme was visualized by reaction with rabbit primary antibodies to RecC and goat anti-rabbit secondary antibodies fused to a fluorescent dye. Cells had a chromosomal *recBCD* deletion and a plasmid with the indicated *recBCD* mutation. *recBCD*$^{+}$ cells contain primarily RecBCD heterotrimer, and *recBC* cells contain primarily RecBC heterodimer, as indicated in the left margin. Purified RecBCD, after long storage, contains about equal amounts of both forms.

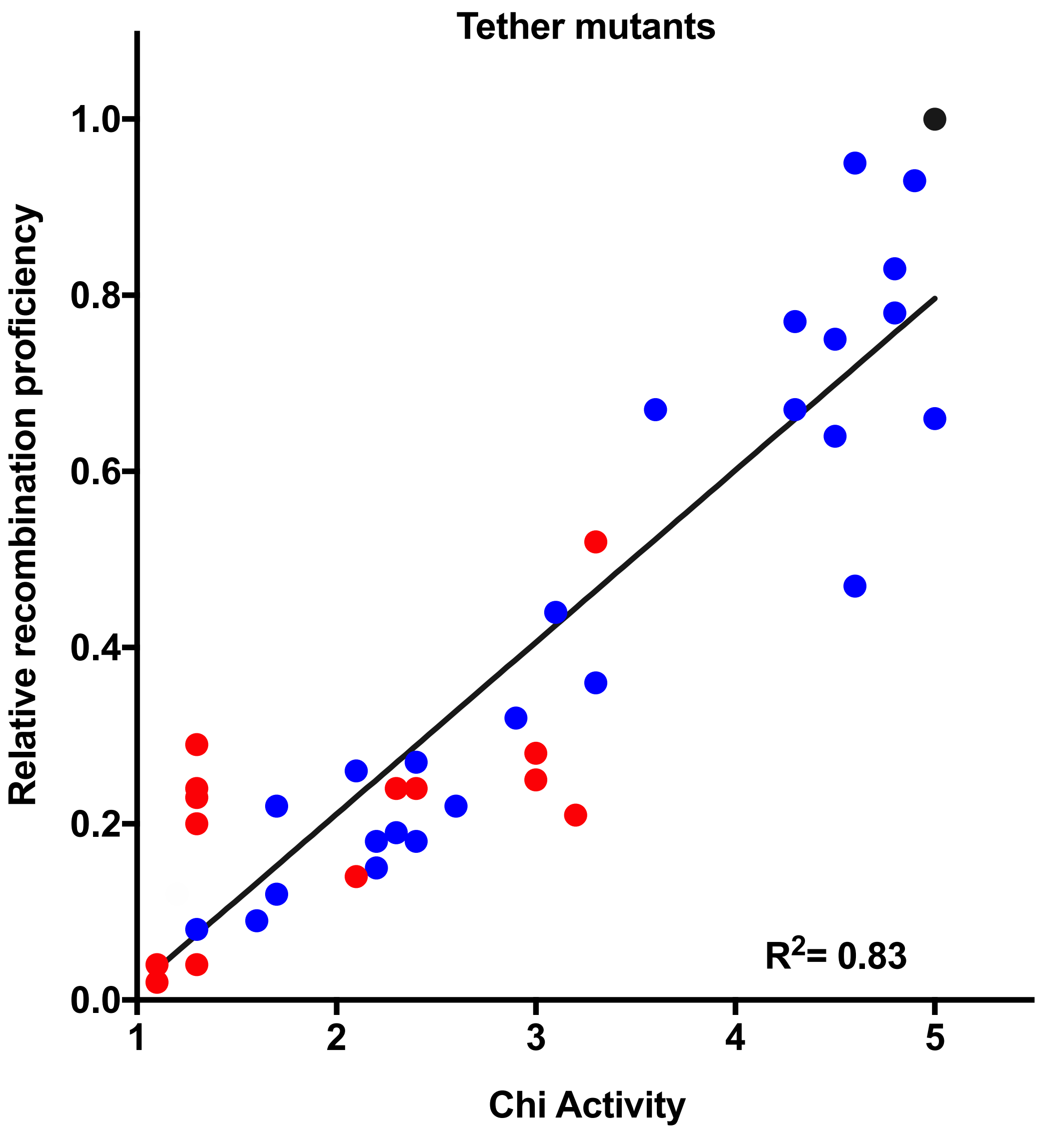

Supplementary Fig. S5. ***E. coli*** **Hfr recombination proficiency is positively correlated with Chi hotspot activity in RecC tether mutants.** Compare with Figure 3 of the main text. Red data points are for deletion mutants and blue for substitution mutants; black point is wild type. $R^2 = 0.83$ ($p < 0.0001$). Modified from [40].

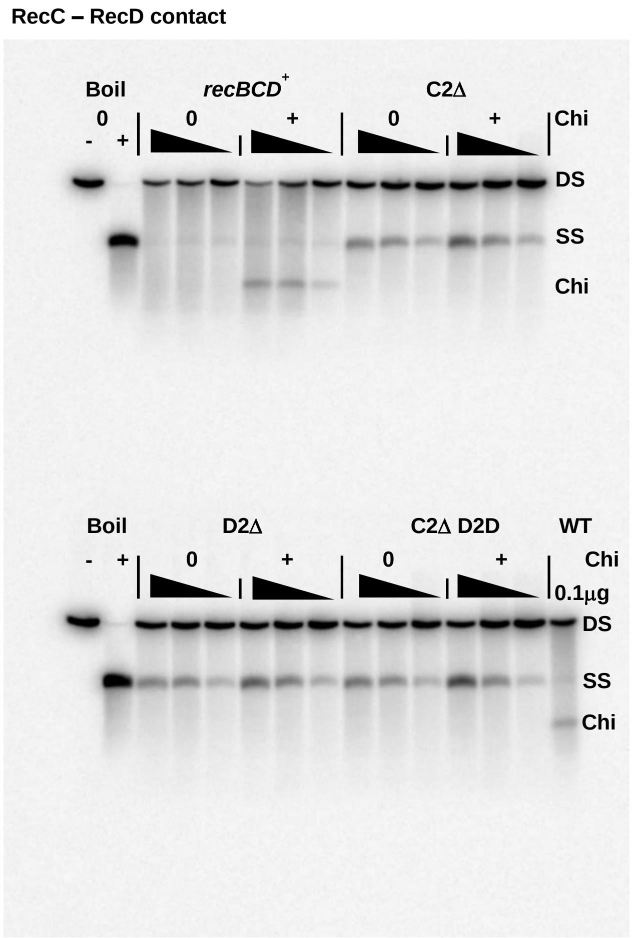

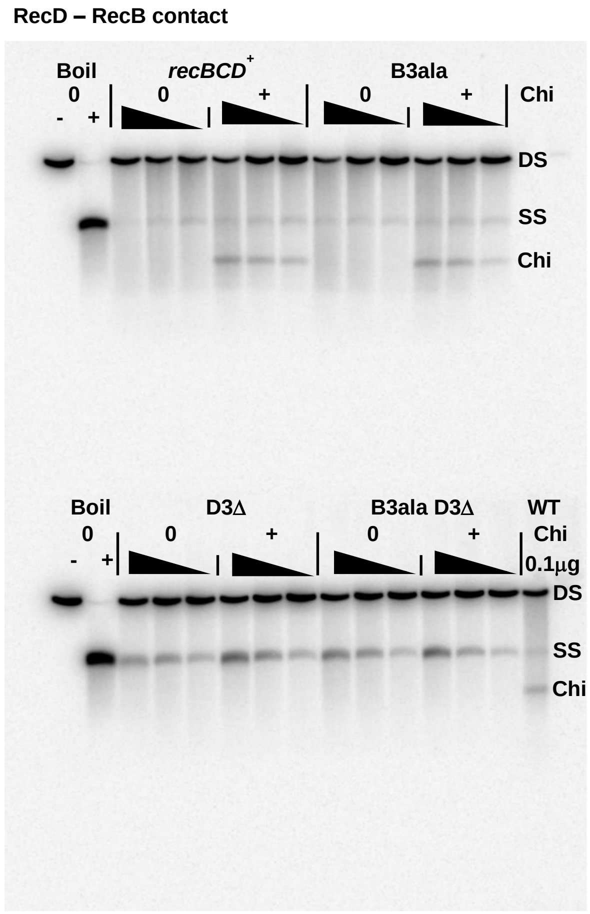

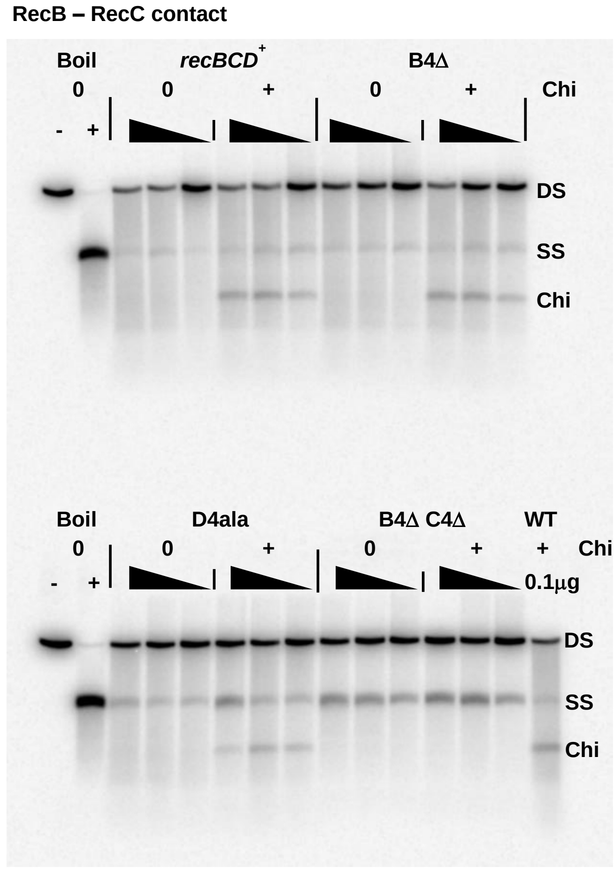

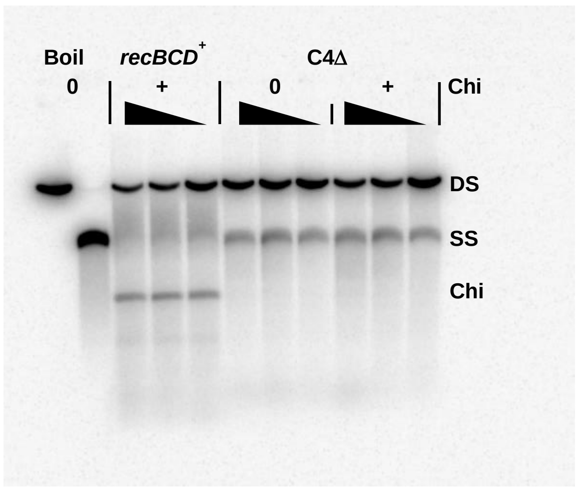

Supplementary Figure S6. RecBCD contact-point mutants retain DNA unwinding but have reduced or undetectable cutting of DNA at Chi hotspots. Shown are full-length blots of the gels corresponding to the data in Figure 4.

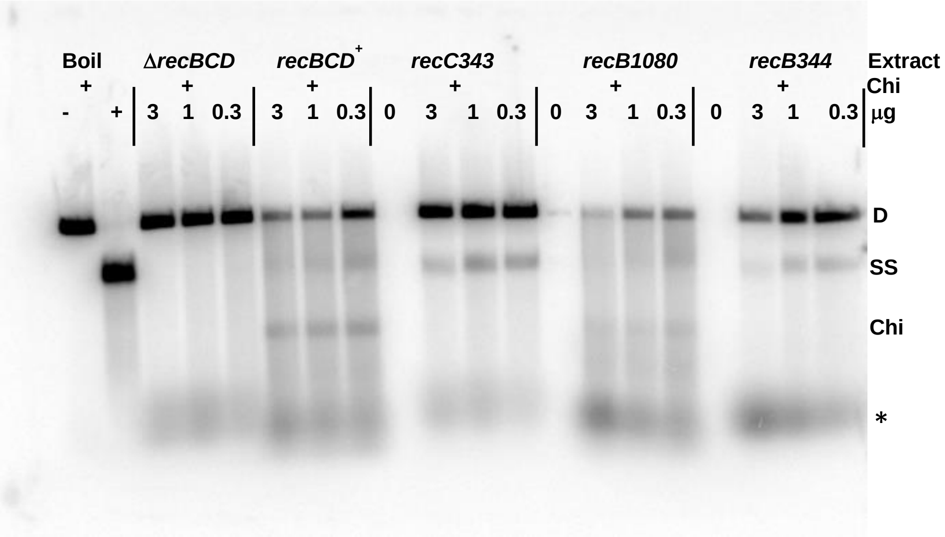

Contact point CD

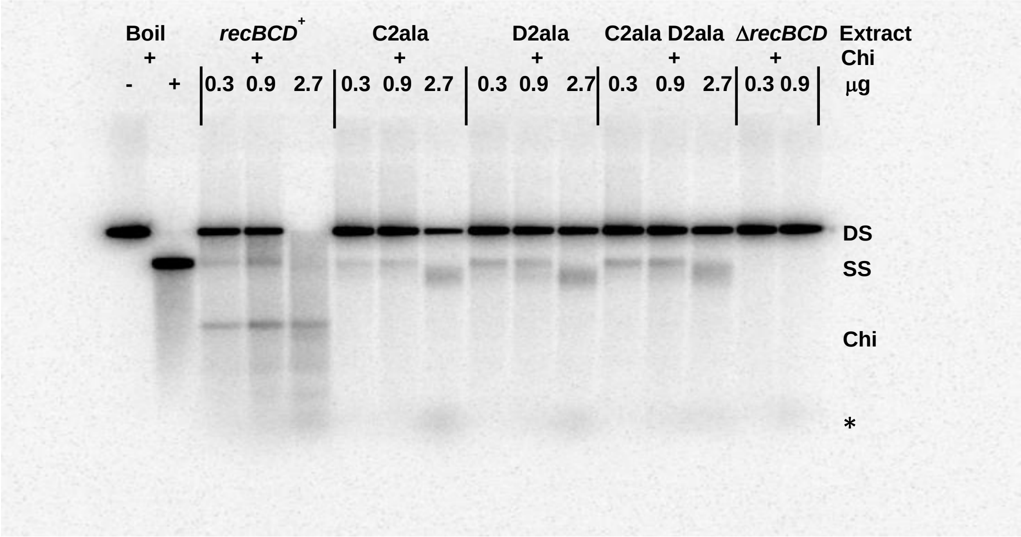

Contact points DB and BC

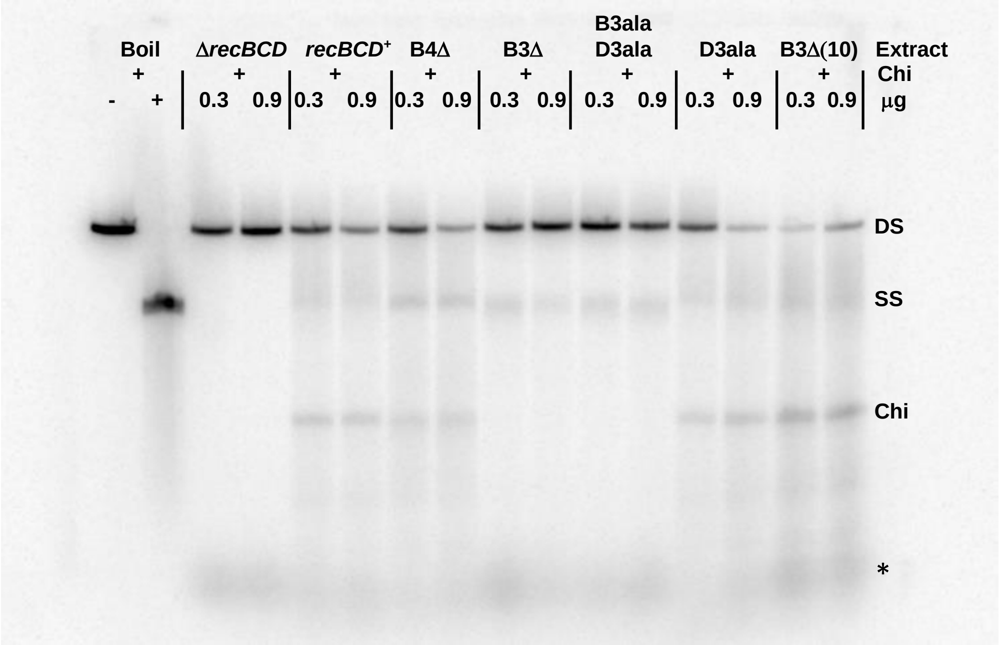

Supplementary Figure S7. RecBCD contact-point mutants, *recC343,* and *recB344* retain DNA unwinding activity but have reduced or undetectable cutting of DNA at Chi hotspots. Shown are full-length blots of the gels corresponding to the data in Supplementary Figure S1.
